# Long COVID prevalence and impact on quality of life 2 years after acute COVID-19

**DOI:** 10.1038/s41598-023-36995-4

**Published:** 2023-07-11

**Authors:** Yoonjung Kim, Sohyun Bae, Hyun-Ha Chang, Shin-Woo Kim

**Affiliations:** Division of Infectious Diseases, Department of Internal Medicine, School of Medicine, Kyungpook National University Hospital, Kyungpook National University, 130, Dongdeok-ro, Jung-gu, Daegu, 41944 Republic of Korea

**Keywords:** Infectious diseases, Virology

## Abstract

There has been an increasing interest in the long-term impact of long COVID. However, only a few studies have investigated the clinical manifestations of long COVID 24 months after acute COVID infection. In this study, prospective online surveys were conducted in adults previously diagnosed with coronavirus disease 2019 (COVID-19) in South Korea between February 13 and March 13, 2020, at 6, 12, and 24 months after COVID-19. We investigated self-reported symptoms and the EuroQol-5-dimension index. Among 900 individuals enrolled initially, 150 completed all 3 surveys. After excluding the cases of COVID-19 reinfection, 132 individuals were included in the final analysis. Among the 132 participants, 94 (71.2%) experienced symptoms of long COVID. The most frequently reported symptoms were fatigue (34.8%), amnesia (30.3%), concentration difficulties (24.2%), insomnia (20.5%), and depression (19.7%). Notably, no significant differences were noted in the incidence of long COVID at 24 months in terms of the number of vaccinations received. Although the neuropsychiatric quality of life improved over time, it continued to affect 32.7% of participants. Symptoms of long COVID, particularly neuropsychiatric symptoms, tend to persist over time, and COVID-19 vaccination or the number of vaccinations received may not significantly affect the incidence of long COVID.

## Introduction

The coronavirus disease 2019 (COVID-19) pandemic has been ongoing for more than 2 years, and the number of cases continues to increase worldwide due to the constant mutation of the virus, evolution of infectivity, and evasion of immunity. Nevertheless, the proportion of people surviving COVID-19 has substantially increased compared to the initial outbreak owing to the improvements in treatment methods and preventive vaccination strategies^1^.

Severe acute respiratory syndrome coronavirus-2 (SARS-CoV-2), the causative agent of COVID-19, is often associated with long-term symptoms known as post-COVID-19 condition or long COVID^2^. While COVID-19 primarily affects the respiratory system, evidence from the relevant literature indicates that it also affects the digestive, cardiovascular, nervous, and reproductive systems^3,4^. According to the World Health Organization (WHO), long COVID is defined as the continuation or development of new symptoms 3 months after the initial SARS-CoV-2 infection, with these symptoms lasting for at least 2 months with no other explanation^5^. Although the symptoms of long COVID typically resolve in most cases^6^, neuropsychiatric symptoms can persist for more than 6–12 months after acute COVID-19^7–10^. This is a significant concern as a previous study suggested similarities between brain changes occurring during and after COVID-19 and those seen in human neurodegenerative diseases^11^. Although risk factors for long COVID include old age, female sex, and moderate or severe COVID-19^12^, long COVID can develop regardless of disease severity^13^. Moreover, long COVID is associated with poor health-related quality of life, both in patients with moderate or severe COVID-19 and in those with mild or asymptomatic COVID-19^14^. Notably, a population-representative survey in the United States reported a high incidence of long COVID (30.2% [18.5–41.8%] within the past 1–6 months)^15^. However, further long-term follow-up studies are necessary to establish a treatment plan for long COVID.

Although some studies have reported that long COVID persists for 24 months after COVID-19^14,16^, there is still a limited number of long-term follow-up studies. Moreover, it has been reported that vaccination before or after COVID-19^13–15^ reduces the risk of developing long COVID^17^. However, to the best of our knowledge, no study has analyzed long-term COVID-19 including the presence or absence of reinfection and the history of vaccination after COVID-19 to date. Therefore, this study aimed to comprehensively assess the longitudinal progression of health outcomes in COVID-19 survivors who did not experience reinfection up to 24 months after acute infection and evaluate the impact of long COVID on the daily lives of such patients.

## Methods

### Study design and population

In this study, three prospective online surveys were conducted with patients diagnosed with COVID-19 (age 16–70 years) from February 13 to March 13, 2020 in Daegu using the clinical data provided by the Daegu Infectious Diseases Control and Prevention. These patients were diagnosed via real-time reverse transcription polymerase chain reaction using nasopharyngeal swabs or other upper respiratory tract samples.

The online survey was conducted at Kyungpook National University Hospital, Daegu. The first survey was conducted at 5–6 months after the onset of COVID-19 symptoms or COVID-19 diagnosis between September 8 and September 10, 2020. The second survey was conducted at 11–12 months after COVID-19 symptom onset or diagnosis between May 26 and June 1, 2021. The third survey was conducted 24 months after COVID-19 symptom onset or diagnosis between May 26 and June 2, 2022. Sex, birth year and date, and the last four digits of cellular phone numbers were used to distinguish and match the responders of the first and second online surveys with those of the third survey.

Long COVID was defined as the presence of at least one sequelae symptom based on the case definition of long COVID^2^, confirmed intermittently or continuously. Participants were considered in the no symptom group if they no longer experienced symptoms at the time of completion of the survey. Responders who were found to be reinfected based on the documented data were excluded.

The individualized questionnaire list included questions related to sex, birth year and date, residential address, COVID-19 diagnosis date, symptoms categorized according to the 45 symptom classification during or after acute COVID-19, oxygen treatment history, admission place during acute COVID-19, vaccination history after COVID-19, newly diagnosed diseases after COVID-19, and outpatient treatment history for COVID-19-related persistent symptoms or signs. Clinical data were obtained from the Daegu Center for Infectious Diseases Control and Prevention registry for COVID-19 diagnosis date, first symptom onset date, disease severity, and reinfection history.

The EuroQol-5 dimension (EQ5D) index was used to assess the quality of life associated with long COVID. The EQ5D score was classified into five categories: mobility, self-care, usual activities (e.g., work, study, housework, family, or leisure activities), pain/discomfort, and anxiety/depression. Further, each category had five levels indicating the severity of problems (no, slight, moderate, severe, and extreme problems). Respondents indicated their health status by selecting the most appropriate statement for each category. The scores for five categories were then combined to form a five-digit number representing the respondent’s health status^18^.

The severity scores during acute COVID-19 were classified as follows: (1) asymptomatic, no symptoms or discomfort throughout the disease period, with a body temperature of < 37.5 °C; (2) mild, presence of symptoms with or without fever (≥ 37.5 °C) but no manifestation or identification of pneumonia; (3) moderate, pneumonia diagnosed by a clinician but not requiring oxygenation therapy other than room air; (4) severe, pneumonia diagnosed by a clinician and requiring oxygenation therapy (nasal prong, facial mask, or high-flow oxygen therapy); and (5) critical, pneumonia diagnosed by a clinician and requiring mechanical ventilation therapy or extracorporeal membrane oxygenation therapy^8^.

### Statistical analysis

We conducted a descriptive analysis. Continuous variables were presented as median (interquartile range [IQR]) values, whereas categorical variables were presented as numbers (percentages). Categorical variables were analyzed using Fisher’s exact or chi-square test, whereas noncategorical variables were analyzed using Student’s *t* or Mann–Whitney U test. Notably, these tests were used to determine the differences in the clinical characteristics and lifestyle changes of the responders with and without long COVID after 24 months of acute COVID-19 and to identify the differences in the prevalence of long COVID over time (from 6 to 24 months) after acute COVID-19. Multivariate logistic regression analysis was performed to determine the potential factors associated with major long COVID symptoms 24 months after the infection. Further, adjusted odds ratios (ORs) with 95% confidence intervals were calculated. A *P*-value of < 0.05 was considered statistically significant. Statistical analyses were performed using R statistics version 4.0.2 (The R Foundation; https://www.r-project.org).

### Ethics approval statement

This study was reviewed and approved by the Institutional Review Board of Kyungpook National University Hospital (approval no.: 2021-02-003). All methods were performed in accordance with the relevant guidelines and regulations by including a statement in the methods section. All respondents provided digital informed consents before the questionnaire was administered.

## Results

### Demographics and characteristics of long COVID for 24 months

Among the 5,252 individuals, 900 responded to the first online survey, and 241 responded to the second online survey. Overall, 150 respondents completed all three surveys, resulting in a total response rate was 62.2% (150 out of 241 respondents). Of these 150 respondents, 18 were excluded due to reinfection. Finally, 132 respondents were included in the analysis. The median number of days from diagnosis to the date of survey was 819 (IQR 816–823). Among the respondents, 90 (68.2%) were females, representing the majority.

The median age of participants at COVID-19 diagnosis was 38 (IQR 24.0–50.5) years, and the age range of 17–29 years was the most common (38.6%). Overall, 26.5% respondents were aged > 50 years. No significant differences were found in sex and age distribution between the groups with and without 24-month long COVID symptoms (*P* > 0.05) (Supplementary Table 1).

Overall, 77.3% of respondents belonged to the mild COVID-19 group, while 15.9% had moderate or severe COVID-19.

Sixteen respondents (12.1%) were diagnosed with new conditions after acute COVID-19, and eight respondents (6.1%) sought outpatient treatment for long COVID symptoms. Notably, all respondents were vaccinated after acute COVID-19. Among the 132 respondents, 127 (96.2%) were vaccinated, and there were no differences in the number of vaccinations between the two groups (Table 1).

**Table 1:** Clinical characteristics of 132 respondents according to the presence of persistent symptoms or signs identified 24 months after the diagnosis or symptom onset of acute COVID-19.

Characteristics	No symptom (N = 38)	Symptom (N = 94)	Total (N = 132)	*P*-value
Days from COVID-19 diagnosis to survey	820.0 [816.0;821.0]	819.0 [816.0;823.0]	819.0 [816.0;823.0]	0.493
Sex				0.159
Male	16 (42.1%)	26 (27.7%)	42 (31.8%)	
Female	22 (57.9%)	68 (72.3%)	90 (68.2%)	
Age, median (IQR; years)	29.5 [23.0;47.0]	40.0 [25.0;52.0]	38.0 [24.0;50.5]	0.097
Age distribution (years)				0.295
18–29	19(50.0%)	32(34.0%)	51 (38.6%)	
30–39	4 (10.5%)	15 (16.0%)	19 (14.4%)	
40–49	9 (23.7%)	18 (19.1%)	27 (20.5%)	
50–59	4 (10.5%)	19 (20.2%)	23 (17.4%)	
60–70	2 (5.3%)	10 (10.6%)	12 (9.1%)	
Age (years)				0.119
< 50	32 (84.2%)	65 (69.1%)	97 (73.5%)	
≥ 50	6 (15.8%)	29 (30.9%)	35 (26.5%)	
Disease severity				0.002
Asymptomatic	7 (18.4%)	2 (2.1%)	9 (6.8%)	
Mid	29 (76.3%)	73 (77.7%)	102 (77.3%)	
Moderate	2 (5.3%)	17 (18.1%)	19 (14.4%)	
Severe	0 (0.0%)	2 (2.1%)	2 (1.5%)	
Critical	0 (0.0%)	0 (0.0%)	0 (0.0%)	
Disease severity				0.062
< Moderate	36 (94.7%)	75 (79.8%)	111 (84.1%)	
≥ Moderate	2 (5.3%)	19 (20.2%)	21 (15.9%)	
Isolation place^a^				0.046
Secondary or tertiary hospital	14 (36.8%)	57 (60.6%)	71 (53.8%)	
Therapeutic living center	22 (57.9%)	34 (36.2%)	56 (42.4%)	
Self-home isolation	2 (5.3%)	3 (3.2%)	5 (3.8%)	
Oxygen treatment				0.626
Yes	0 (0.0%)	2 (2.1%)	2 (1.5%)	
No	38 (100.0%)	92 (97.9%)	130 (98.5%)	
Newly diagnosed diseases following acute COVID-19				0.016
Yes	0 (0.0%)	16 (17.0%)	16 (12.1%)	
No	38 (100.0%)	78 (83.0%)	116 (87.9%)	
Outpatient clinic visits due to long COVID				0.146
Yes	0 (0.0%)	8 (8.5%)	8 (6.1%)	
No	38 (100.0%)	86 (91.5%)	124 (93.9%)	
COVID-19 vaccination history^b^				1.000
Yes	37 (97.4%)	90 (95.7%)	127 (96.2%)	
No	1 (2.6%)	4 (4.3%)	5 (3.8%)	
Number of COVID-19 vaccination				1.000
< 2	2 (5.3%)	5 (5.3%)	7 (5.3%)	
≥ 2	36 (94.7%)	89 (94.7%)	125 (94.7%)	
Number of COVID-19 vaccination				0.454
0	1 (2.6%)	4 (4.3%)	5 (3.8%)	
1	1 (2.6%)	1 (1.1%)	2 (1.5%)	
2	10 (26.3%)	26 (27.7%)	36 (27.3%)	
3	18 (47.4%)	31 (33.0%)	49 (37.1%)	
4	8 (21.1%)	32 (34.0%)	40 (30.3%)	

*COVID-19* coronavirus disease 2019, *IQR* interquartile range.

^a^Isolation place: the place where patients were quarantined after being diagnosed with COVID-19 for the first time.

^b^All respondents had received COVID-19 vaccination following acute COVID-19.

### The prevalence of long COVID after 24 months of acute COVID-19

Out of the 132 respondents, 79 (59.8%) reported experiencing one or more long COVID symptoms at 6 months after acute COVID-19, and the most common symptoms were cognitive dysfunction (26.5%), amnesia (25.8%), depression (25.0%), anxiety (24.2%), and concentration difficulties (23.5%).

At 12 months after acute COVID-19, 70 (53.0%) respondents reported experiencing one or more long COVID symptoms, and the most common symptoms at this time point were cognitive dysfunction (23.5%), concentration difficulties (22.0%), amnesia (21.2%), fatigue (17.4%), and anxiety (15.9%).

**Figure 1:**
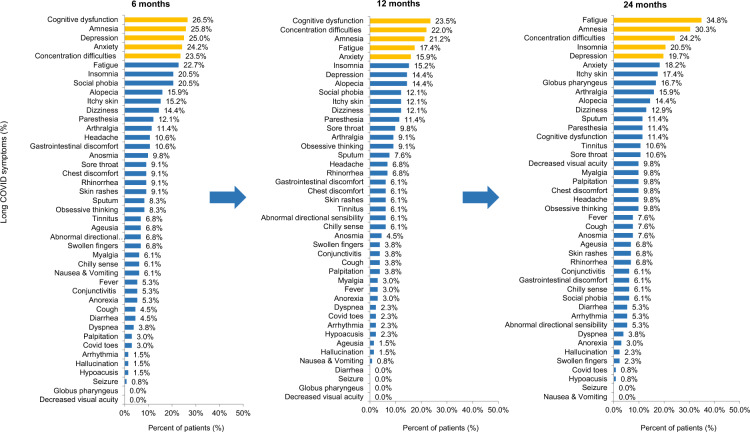
Distribution of 45 persistent symptoms or signs at 6, 12, and 24 months after acute COVID-19.

At 24 months after acute COVID-19, 94 (71.2%) respondents reported experiencing one or more long COVID symptoms. Fatigue (34.8%) was the most frequent symptom, followed by amnesia (30.3%), concentration difficulties (24.2%), insomnia (20.5%), and depression (19.7%) (Fig. 1).

**Figure 2:**
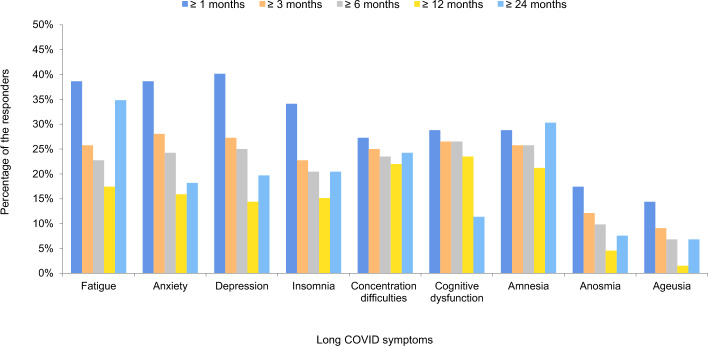
Duration of key long COVID symptoms according to the symptom persistent period at 1, 3, 6, 12, and 24 months after acute COVID-19.

Three (2.3%) respondents exhibited long COVID at 24 months after COVID-19, which was not identified at 12 months after COVID-19. Follow-up examinations at 6, 12, and 24 months from the date of COVID-19 diagnosis or symptom onset showed a high incidence of symptoms at 6 and 12 months after COVID-19, whereas the symptoms remained at the same level even after 24 months. After 6 months, there was no incidence of seizures; however, the incidence of symptoms related to amnesia increased to 30.3%, which was higher than the rates reported at each previous time point. Amnesia, concentration difficulties, alopecia, dizziness, and paresthesia were identified in 15–30% of respondents even 24 months after acute COVID-19 (Fig. 1, Supplementary Figs. 1, 2). In particular, changes in concentration difficulties and amnesia from 1 to 24 months after the date of diagnosis or symptom onset remained at a similar level (25–30%) until 24 months after acute COVID-19 (Fig. 2).

Statistical analysis revealed that the prevalence of fatigue increased over time as cognitive impairment improved (*P* = 0.007). However, no significant changes were found in the other seven symptoms. Moreover, no statistically significant difference was confirmed in the increase or decrease in the prevalence of the other symptoms over time (*P* > 0.05; Supplementary Fig. 3).

Sankey flow diagram analysis was used to assess the frequency of changes in the nine main symptoms from their initial stages over time. The results revealed that these neuropsychiatric symptoms continued to be associated with each other over time at 6, 12, and 24 months (Supplementary Fig. 4).

### The impact of long COVID on quality of life and lifestyle changes

The distribution of EQ5D-5L average scores at 12 and 24 months after COVID-19, measured using five EQ5D categories, revealed that the distribution at 12 months after COVID-19 was similar to that at 24 months after the infection (Supplementary Fig. 5).

**Figure 3:**
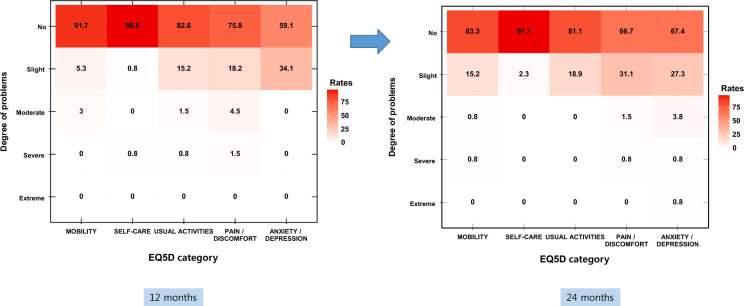
Assessment of quality of life (EQ5D) 12 and 24 months after acute COVID-19 according to the data collected from an online survey involving 132 responders.

Specific answers showed similar distributions in each of the five categories for 12 and 24 months. Thus, it was confirmed that COVID-19 may continue to affect the quality of life of individuals even 24 months after acute infection (Fig. 3).

To determine the effects of long COVID on lifestyle, we compared the lifestyle changes of the participants in the 24-month long COVID symptom presence and absence groups. The results indicated that the long COVID symptom presence group (52.1%) had a higher tendency to consume healthy foods than the absence group (21.1%) (*P* = 0.003). Moreover, the long COVID symptom presence group (36.2%) had a higher tendency to engage in physical activity than the absence group (18.4%); however, no statistical difference was observed between the two groups (*P* = 0.075). Furthermore, no statistical differences were identified between the two groups in terms of smoking or alcohol intake (*P* ≥ 0.05) (Supplementary Table 2).

### Assessing risk factors for long COVID 24 months after acute COVID-19

Risk factor analysis revealed that moderate or severe disease activity during acute COVID-19 is a statistically significant risk factor for long-term symptoms of fatigue (odds ratio, 3.02 [1.17–8.05]; *P* = 0.023). Other symptoms, including anxiety, depression, insomnia, concentration difficulty, and cognitive dysfunction, were not associated with moderate or severe disease activity, age of ≥ 50 years, and sex at the time of acute COVID-19 (*P* > 0.05). The risk of occurrence of amnesia tended to increase by 2.31 times in people aged > 50 years; however, this was not statistically significant (*P* = 0.059) (Table 2).

**Table 2:** Univariate and multivariate logistic regression analyses for the factors associated with long COVID 24 months following COVID-19.

Persistent symptoms or signs	Factors	Univariate OR		Multivariate OR	
		95% CI	*P*-value	95% CI	*P*-value
Fatigue	≥ Moderate severity	3.02 (1.17–8.05)	0.023	3.02 (1.17–8.05)	0.023
	≥ 50 years old	1.35 (0.60–3.00)	0.456	–	–
	Female	0.95 (0.44–2.06)	0.887	–	–
Anxiety	≥ Moderate severity	2.07 (0.66–5.87)	0.185	2.07 (0.66–5.87)	0.185
	≥ 50 years old	1.18 (0.42–3.04)	0.745	–	–
	Female	0.92 (0.37–2.46)	0.86	–	–
Depression	≥ Moderate severity	1.82 (0.59–5.11)	0.270	1.85 (0.55–5.77)	0.297
	≥ 50 years old	1.30 (0.49–3.26)	0.587	1.30 (0.49–3.26)	0.885
	Female	1.34 (0.53–3.70)	0.551	1.34 (0.53–3.70)	0.478
Insomnia	≥ Moderate severity	1.71 (0.56–4.79)	0.319	–	–
	≥ 50 years old	2.32 (0.94–5.65)	0.065	2.32 (0.94–5.65)	0.065
	Female	1.14 (0.46–2.99)	0.784	–	–
Concentration difficulty	≥ Moderate severity	2.23 (0.80–5.94)	0.112	1.80 (0.60–5.14)	0.281
	≥ 50 years old	2.01 (0.84–4.70)	0.109	1.69 (0.66–4.19)	0.264
	Female	1.26 (0.54–3.15)	0.607	–	–
Cognitive dysfunction	≥ Moderate severity	2.14 (0.54–7.11)	0.235	1.70 (0.39–6.37)	0.451
	≥ 50 years old	2.02 (0.63–6.11)	0.216	1.71 (0.48–5.63)	0.382
	Female	0.93 (0.31–3.14)	0.874	0.98 (0.32–3.40)	0.969
Amnesia	≥ Moderate severity	1.94 (0.73–5.04)	0.177	1.54 (0.52–4.42)	0.424
	≥ 50 years old	2.56 (1.14–5.79)	0.023	2.31 (0.96–5.55)	0.059
	Female	1.93 (0.84–4.74)	0.133	2.07 (0.88–5.28)	0.109
Anosmia	≥ Moderate severity	4.12 (0.97–15.99)	0.042	2.45 (0.51–10.70)	0.238
	≥ 50 years old	4.81 (1.29–19.94)	0.021	3.61 (0.85–16.23)	0.080
	Female	1.95 (0.46–13.33)	0.411	–	–
Ageusia	≥ Moderate severity	4.99 (1.14–20.75)	0.026	2.72 (0.55–12.69)	0.203
	≥ 50 years old	6.48 (1.61–32.26)	0.011	4.70 (1.02–25.25)	0.051
	Female	1.69 (0.39–11.67)	0.526	–	–

*CI* confidence interval, *COVID-19* coronavirus disease 2019, *OR* odds ratio.

## Discussion

To the best of our knowledge, this is the first study to assess the long-term impact of COVID-19 at 24 months after acute COVID-19 in patients with documented COVID-19 vaccination and without history of reinfection. Based on the findings of this study, long COVID symptoms improved over time and neuropsychiatric symptoms tended to persist longer than other symptoms up to 24 months after COVID-19.

The clinical spectrum of long COVID comprises a wide range of symptoms. A previous systematic review reported that common symptoms of long COVID include fatigue, dyspnea, concentration impairment, anxiety, sleep disorder, memory impairment, and cognitive impairment^4^. According to the United Kingdom (UK) Office for National Statistics survey, fatigue continued to be the most commonly reported symptom among individuals with long COVID (70% of patients with self-reported long COVID), followed by difficulty concentrating (48%)^19^. Furthermore, in China, a follow-up study on long COVID in 4% of all patients admitted to the intensive care unit confirmed that fatigue was the most common symptom^14^. Additionally, a previous study suggested that postviral somatic and mental symptoms had neuroimmune and neuro-oxidative origins^20^. In the present study, fatigue and mental symptoms accounted for the majority of long COVID symptoms at 24 months after acute COVID-19.

According to a previous study, the risk of mood and anxiety disorders subsides 1–2 months after infection, whereas the risk of psychotic disorders, cognitive deficits, and dementia may remain high even 2 years after COVID-19^21^. In the present study, compared with the other symptoms, the incidence of concentration difficulties and amnesia remained high even after 24 months of infection, similar to the incidence after the occurrence of initial symptoms. However, compared with 1 month after COVID-19, the symptoms of depression and anxiety improved by > 50% at 24 months after COVID-19. Moreover, significant improvement was noted in cognitive dysfunction at 24 months after COVID-19. Thus, it is difficult to exclude the possibility of the effects of mood disorders on cognitive dysfunction due to the tendency of these cognitive dysfunctions to decrease with the improvement in mood disorder. Major depression has commonly been associated with cognitive problems^22^. Moreover, it has been reported that depressive psychopathology most commonly affects cognitive performance as it interacts with cognitive functions in determining the quality of life^23^. Additionally, Sankey flow diagrams showed correlations among neuropsychological symptoms.

In a previous study, 1 year after acute COVID-19, long COVID developed more frequently in patients with moderate or higher illness severity than in asymptomatic patients^24^. A prospective observational study of post-COVID-19 chronic fatigue syndrome reported that it was associated with symptom severity^25^. Previous studies involving 24 month-follow-ups of patients with COVID-19 have suggested that patients with severe disease activity are 1.45 and 1.54 times more likely to develop fatigue or muscle weakness and anxiety or depressive symptoms, respectively^14^. Moreover, moderate or higher disease severity during acute COVID-19 was identified as a risk factor, with an odds ratio of 3.02 for long-term fatigue symptoms 24 months after acute COVID-19. However, in our study, anxiety or depressive symptoms were not associated with disease activity 24 months after acute COVID-19.

Notably, individuals with long COVID symptoms for 2 years exhibited poor health-related quality of life (HRQoL), high mental health abnormality, and increased healthcare use after discharge compared to those without long COVID symptoms. Moreover, previous studies reported that the quality of life may worsen during follow-up for COVID-19^26^, and compared to participants without COVID-19, COVID-19 survivors continued to exhibit higher symptom prevalence with more pain or discomfort and anxiety or depressive symptoms 2 years after the infection. However, their HRQoL continued to improve for nearly all domains, particularly for anxiety or depression, with the proportion of participants reporting anxiety or depressive symptoms decreasing significantly from 23 at 6 months to 12% at 2 years after COVID-19^14^. In the present study, compared with the ability to perform usual activities and pain/discomfort of the patients at 12 months after COVID-19, those at 24 months after the infection improved. However, the number of people with reduced quality of life because of anxiety and depressive symptoms did not significantly change (34.1% and 32.7% at 12 and 24 months after COVID-19, respectively). In addition, 1.6% of the respondents who stated that they experienced severely reduced quality of life because of anxiety and depressive symptoms were newly identified. Unlike a previous study with a median participant age of 57 years^14^, our study included young people with a median age of 38 years, suggesting that the younger age group is more affected by COVID-19 and long COVID symptoms than the older age group. This finding is consistent with that of a previous study reporting that the risk of developing long COVID increases with age^27^. Other factors affecting the decrease in HRQoL could be attributed to the emotional and social factors related to the COVID-19 outbreak depending on the situation of each country^14^. Additionally, social isolation due to the long-lasting COVID-19 pandemic can be a major contributor to the depressive symptoms associated with long COVID symptoms or poor quality of life^28^. According to a study on long COVID in patients discharged from COVID-19-treatment specialized hospitals in Korea, the patients considered it challenging to return to normal life owing to the presence of post-COVID-19 symptoms. In particular, returning to daily life was found to be difficult for older people^29^. In the present study, 6.1% of respondents were receiving outpatient treatment for long COVID, which consequently affected their daily quality of life. The present study included patients exhibiting mild disease severity during acute illness. The findings revealed that long COVID could persist for a long time in patients with COVID-19, suggesting that long COVID is a long-term social burden even in mildly infected patients and can thus account for the majority of patients infected with COVID-19.

The findings of a UK-based survey suggest that post-infection vaccination reduces the symptomatic burden of long COVID after the first dose, with sustained improvement after the second dose^17^. Notably, patients with long COVID and immune system dysregulation may benefit from a reset of autoimmune processes via vaccination (although whether this effect is long-lasting remains unestablished), and any residual viral reservoir may also be destroyed due to the antibody response^30^.

In the present study, 96.2% of respondents were vaccinated after COVID-19. Furthermore, after 24 months, 71.2% of respondents reported > 1 suspected long COVID symptom. Amnesia was identified in 21.2% of respondents at 12 months after COVID-19, and this rate increased to 30.3% at 24 months after the infection. Concentration difficulties were identified in 22.0% of the respondents at 12 months after COVID-19, and this rate increased to 24.2% at 24 months after the infection. Notably, the decreased gray matter in the temporal lobe in patients with COVID-19 highlighted the increased risk of later neurodegeneration and dementia due to viral invasion of the central nervous system^31^. Moreover, previous studies have consistently reported an association between COVID-19 and memory impairment^32,33^. As indicated by the results of the present study, the neuropsychological effects of long COVID can significantly compromise the quality of life of adults exhibiting mild COVID-19. However, the impact of vaccination on people with long-term COVID symptoms remains controversial. Therefore, well-designed research on the effects of COVID-19 vaccination on the improvement or persistence of long-term COVID symptoms after acute COVID-19 and the long-term effects of COVID-19 vaccination is warranted.

This study has a few limitations. First, due to the long-term follow-up period of the study and nonhomogeneity of the patients, there may be limitations in interpreting whether persistent symptoms are related to long COVID. Moreover, other factors such as sociocultural differences may have influenced the results of the surveys. Therefore, further studies on different sociocultural backgrounds are needed to elucidate the effects of long COVID. Second, because of the limitations of the online survey, no individualized tests of cognitive function could be performed for comparison. Thus, further studies using objective cognitive tests must be applied to individuals, and differences should be substantiated by the literature. Third, it is difficult to state that the sample was highly representative of any given population because, at the time of the survey, few respondents had severe or critical disease activity during acute COVID-19, and older adults may have experienced difficulty participating in online surveys. Furthermore, individuals exhibiting long COVID symptoms may have participated more actively in the survey, leading to a relatively high incidence of long COVID among respondents.

The strength of this study is that it revealed the long-term effects of COVID-19 in relatively young patients with mild acute illness after excluding confounding factors and reinfection cases. Remarkably, long COVID symptoms manifested in various forms up to 24 months after acute COVID-19 illness, even in patients who were vaccinated after COVID-19. Furthermore, our results suggest that even 24 months after acute COVID-19, the prevalence of long COVID symptoms and reduced quality of life in patients with COVID-19 is a concern that represents a long-term social burden regardless of the severity of the disease.

## Conclusions

Although long COVID usually improves over time, neuropsychiatric symptoms can persist for up to 24 months after an acute infection and occur more frequently than other symptoms. Patients with mild COVID-19 disease, who account for the majority of patients with COVID-19, may continue to have a poor quality of life. In addition, the occurrence of long COVID does not appear to be significantly affected by COVID-19 vaccination or the number of vaccinations received. Therefore, ongoing research on long COVID is needed to better understand the potential long-term health consequences of COVID-19, including the persistence of symptoms, impact on quality of life, and effectiveness of interventions such as vaccination.

## Supplementary Information

Supplementary Information.

## Data Availability

The datasets generated during and/or analyzed during the current study are available from the corresponding author upon reasonable request.
