# Author Correction: Long COVID prevalence and impact on quality of life 2 years after acute COVID-19

**DOI:** 10.1038/s41598-023-39132-3

**Published:** 2023-07-24

**Authors:** Yoonjung Kim, Sohyun Bae, Hyun-Ha Chang, Shin-Woo Kim

**Affiliations:** Division of Infectious Diseases, Department of Internal Medicine, School of Medicine, Kyungpook National University Hospital, Kyungpook National University, 130, Dongdeok-ro, Jung-gu, Daegu, 41944 Republic of Korea

Correction to: *Scientific Reports* 10.1038/s41598-023-36995-4, published online 11 July 2023

The original version of this article contained an error in the Abstract, where

"There has been an increasing interest in the long-term impact of long COVID. However, only a few studies have investigated the clinical manifestations of long COVID after 24 months of acute infection.”

now reads:

"There has been an increasing interest in the long-term impact of long COVID. However, only a few studies have investigated the clinical manifestations of long COVID 24 months after acute COVID infection.”

The original article has been corrected.

