# Supplementary material for: Long COVID prevalence and impact on quality of life 2 years after acute COVID-19
Source: Sci Rep. 2023 Jul 11;13:11207. doi: 10.1038/s41598-023-36995-4 (PMC10336045; doi:10.1038/s41598-023-36995-4)
Supplement: Supplementary file 1 — Supplementary Information. [file 41598_2023_36995_MOESM1_ESM.docx]

**Enduring Symptoms: The Prevalence and Impact of Long COVID 24 Months After Acute Infection**

Yoonjung Kim, Sohyun Bae, Hyun-Ha Chang, Shin-Woo Kim

Division of Infectious Diseases, Department of Internal Medicine, Kyungpook National University Hospital, School of Medicine, Kyungpook National University, Daegu, Republic of Korea

**Running title:** A 24-month follow-up of long COVID

**Corresponding author**:

Shin-Woo Kim, MD, PhD

Department of Internal Medicine, Kyungpook National University School of Medicine, 130, Dongdeok-ro, Jung-gu, Daegu, 41944, Korea

Tel: +82-53-200-6525

Fax: +82-53-424-5542

E-mail: [ksw2kms@knu.ac.kr](mailto:ksw2kms@knu.ac.kr)

Supplementary Table 1. Sex and age distribution of 132 respondents based on symptoms or signs 24 months following acute COVID-19 infection.

| **Characteristics** | **No symptom** | | | | **Presence of symptom** | | | |
| --- | --- | --- | --- | --- | --- | --- | --- | --- |
| **Sex** | Male | Female | Total | *P*- value | Male | Female | Total | *P*- value |
|  | (N = 16) | (N = 22) | (N = 38) |  | (N = 26) | (N = 68) | (N = 94) |  |
| **Age (years)** |  |  |  | 1.000 |  |  |  | 1.000 |
| **<50** | 13 (81.2%) | 19 (86.4%) | 32 (84.2%) |  | 18 (69.2%) | 47 (69.1%) | 65 (69.1%) |  |
| **≥50** | 3 (18.8%) | 3 (13.6%) | 6 (15.8%) |  | 8 (30.8%) | 21 (30.9%) | 29 (30.9%) |  |
| **Age distribution (years)** |  |  |  | 0.107 |  |  |  | 0.165 |
| **18–29** | 5 (31.2%) | 14 (63.6%) | 19 (50.0%) |  | 5 (19.2%) | 27 (39.7%) | 32 (34.0%) |  |
| **30–39** | 3 (18.8%) | 1 (4.5%) | 4 (10.5%) |  | 7 (26.9%) | 8 (11.8%) | 15 (16.0%) |  |
| **40–49** | 5 (31.2%) | 4 (18.2%) | 9 (23.7%) |  | 6 (23.1%) | 12 (17.6%) | 18 (19.1%) |  |
| **50–59** | 3 (18.8%) | 1 (4.5%) | 4 (10.5%) |  | 4 (15.4%) | 15 (22.1%) | 19 (20.2%) |  |
| **60–70** | 0 (0.0%) | 2 (9.1%) | 2 (5.3%) |  | 4 (15.4%) | 6 (8.8%) | 10 (10.6%) |  |

Supplementary Table 2. Lifestyle-change assessment among the 132 respondents 24 months following acute COVID-19 infection

| **Characteristics** | **No symptom**  **(N = 38)** | **Symptom**  **(N = 94)** | **Total**  **(N= 132)** | **P-value** |
| --- | --- | --- | --- | --- |
| **Smoking** |  |  |  | 0.196 |
| I do this more often | 0 (0.0%) | 0 (0.0%) | 0 (0.0%) |  |
| I do this less often | 1 (2.6%) | 2 (2.1%) | 3 (2.3%) |  |
| No difference | 8 (21.1%) | 9 (9.6%) | 17 (12.9%) |  |
| I did not do this before COVID-19 | 29 (76.3%) | 83 (88.3%) | 112 (84.8%) |  |
| **Drinking alcohol** |  |  |  | 0.110 |
| I do this more often | 1 (2.6%) | 6 (6.4%) | 7 (5.3%) |  |
| I do this less often | 5 (13.2%) | 16 (17.0%) | 21 (15.9%) |  |
| No difference | 22 (57.9%) | 33 (35.1%) | 55 (41.7%) |  |
| I did not do this before COVID-19 | 10 (26.3%) | 39 (41.5%) | 49 (37.1%) |  |
| **Eating healthy food** |  |  |  | 0.003 |
| I do this more often | 8 (21.1%) | 49 (52.1%) | 57 (43.2%) |  |
| I do this less often | 1 (2.6%) | 7 (7.4%) | 8 (6.1%) |  |
| No difference | 24 (63.2%) | 32 (34.0%) | 56 (42.4%) |  |
| I did not do this before COVID-19 | 5 (13.2%) | 6 (6.4%) | 11 (8.3%) |  |
| **Physical activity**  **(including walking & cycling)** |  |  |  | 0.075 |
| I do this more often | 7 (18.4%) | 34 (36.2%) | 41 (31.1%) |  |
| I do this less often | 7 (18.4%) | 21 (22.3%) | 28 (21.2%) |  |
| No difference | 22 (57.9%) | 32 (34.0%) | 54 (40.9%) |  |
| I did not do this before COVID-19 | 2 (5.3%) | 7 (7.4%) | 9 (6.8%) |  |

Abbreviation: COVID-19, coronavirus disease.

Data are presented as n (%).


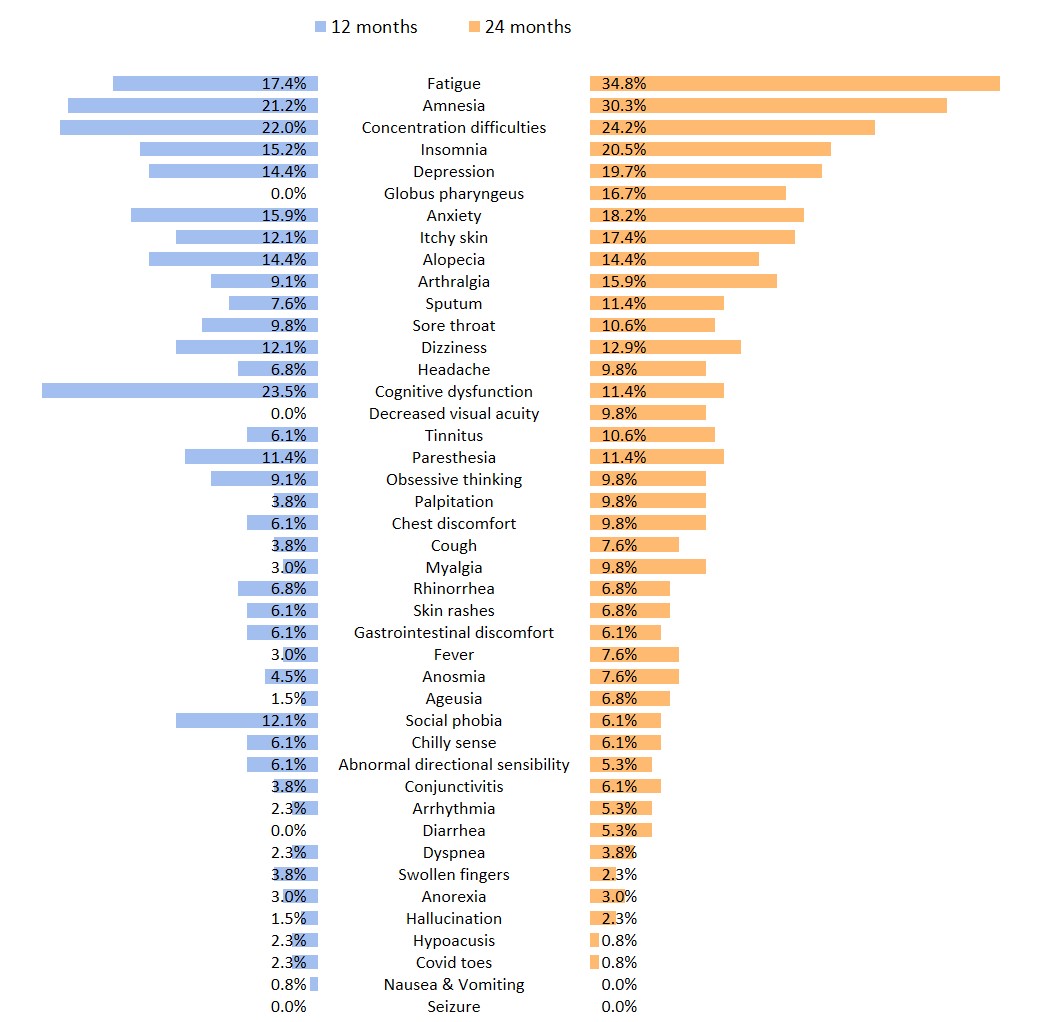


Supplementary Figure 1. Distribution of 45 persistent symptoms or signs at 12 and 24 months following acute COVID-19 infection.


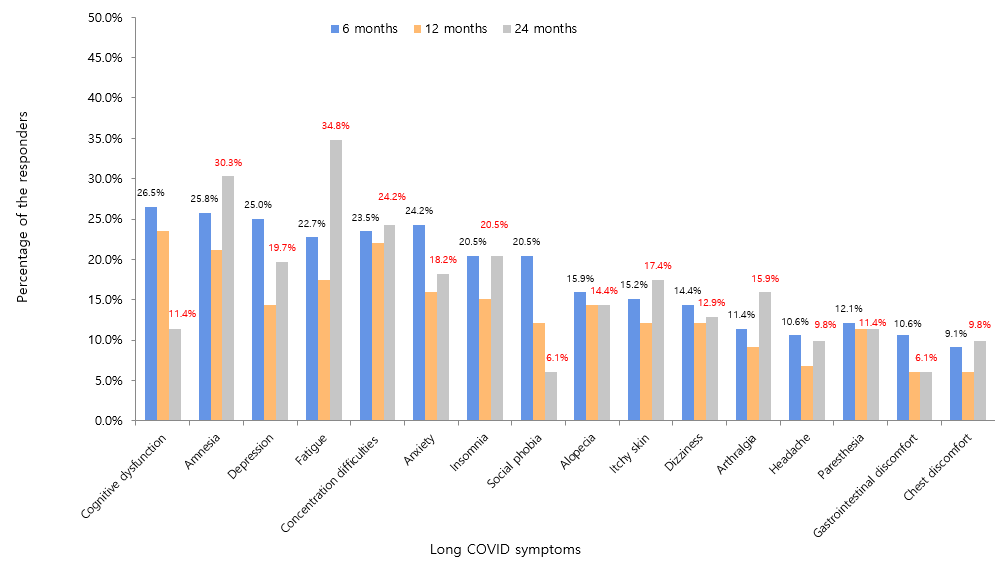


Supplementary Figure 2. Difference in long COVID prevalence overtime at 6, 12, and 24 months following acute COVID-19 infection.


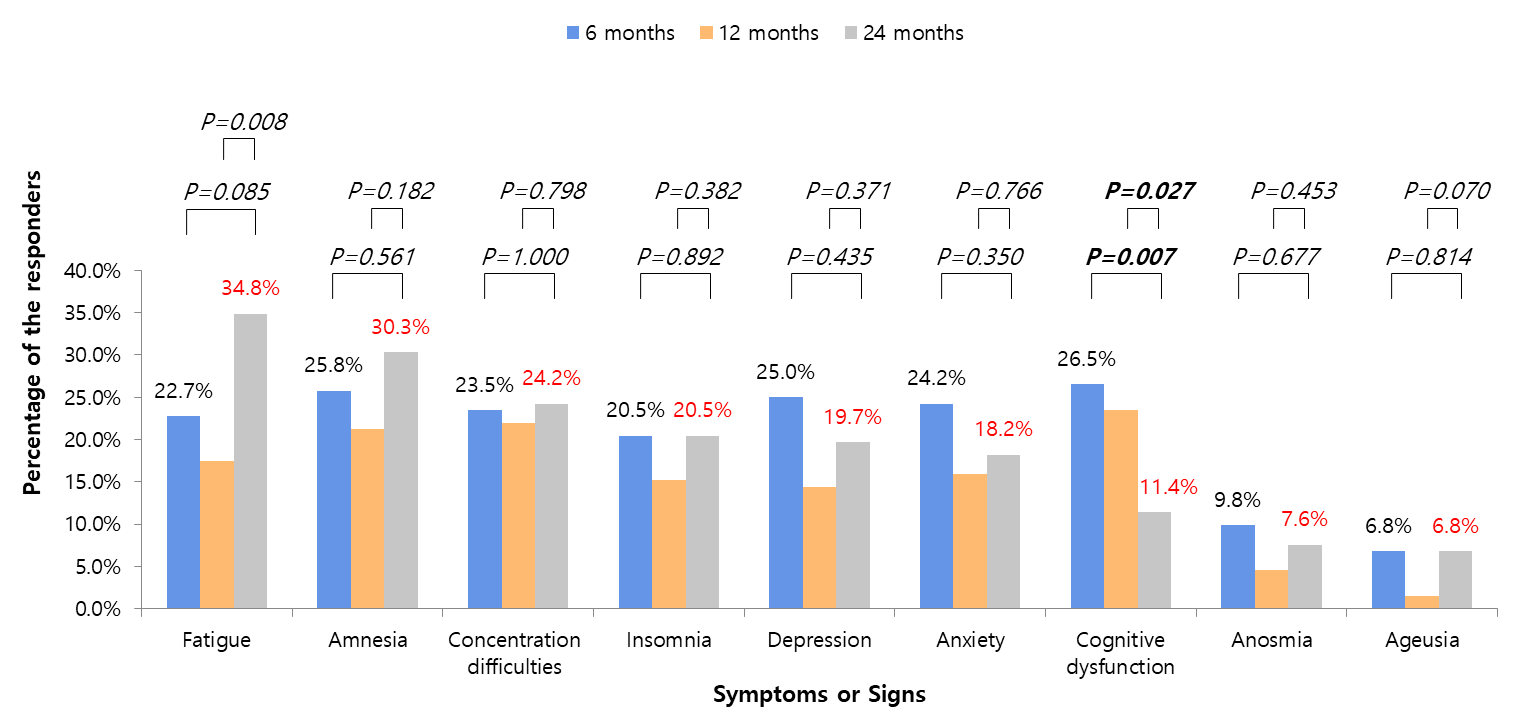


Supplementary Figure 3. Difference in long COVID prevalence over time.


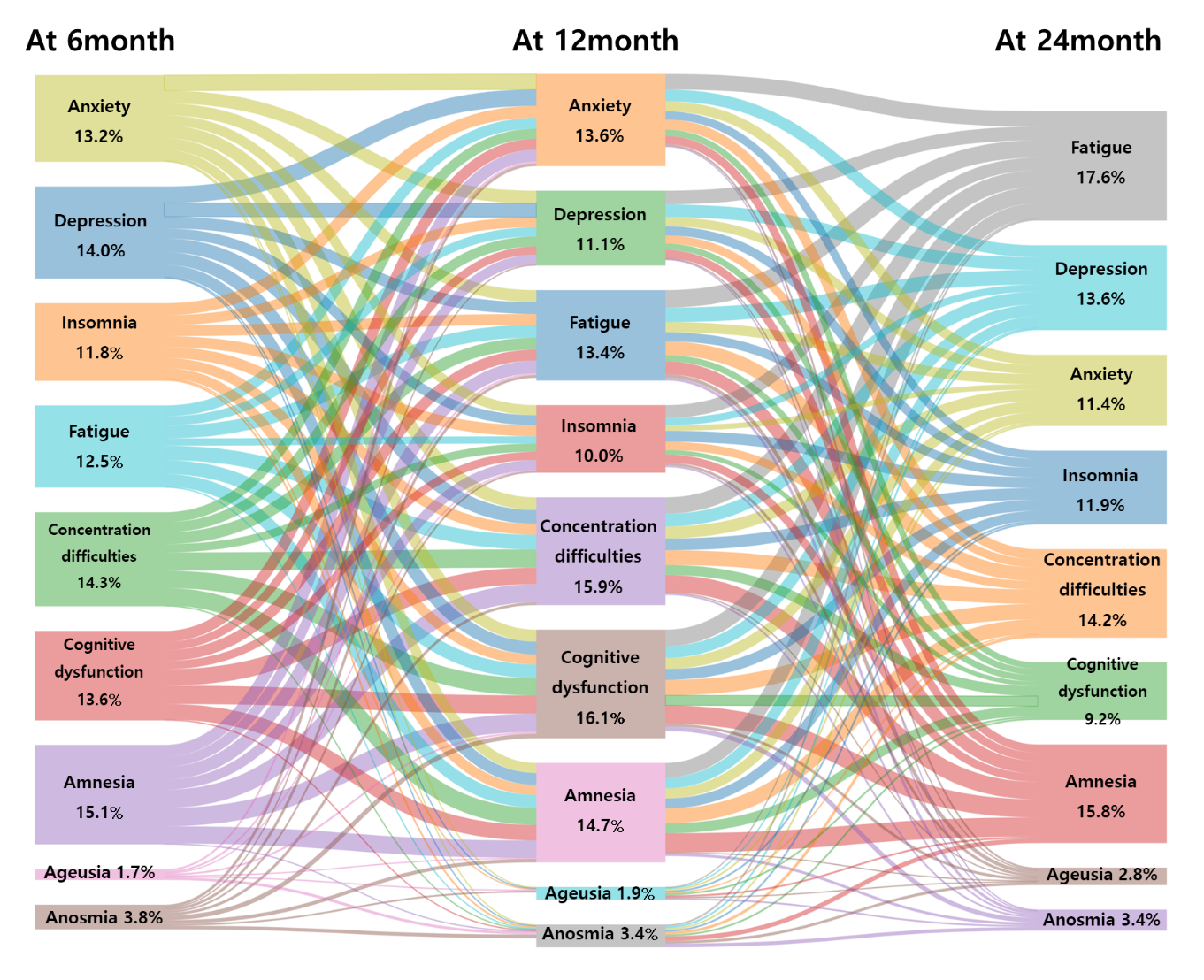


Supplementary Figure 4. Overview of Sankey flow diagrams focusing on long-COVID symptoms and signs in patients with COVID-19 6, 12, and 24 months following the infection from the data collected in an online survey involving 132 responders.

Supplementary Figure 5. Distribution of EQ5D-5L average values at 24 months following

acute COVID-19 infection in 132 respondents. Each domain of EQ-5D is scored on a 5-point scale: 1, no problem; 2, slight problem; 3. moderate problem; 4, severe problem; and 5, unable to do or extreme problem.
